# Mitochondrial membrane synthesis, remodelling and cellular trafficking

**DOI:** 10.1002/jimd.12766

**Published:** 2024-06-14

**Authors:** Martina Messina, Frédéric M. Vaz, Shamima Rahman

**Affiliations:** ^1^ Mitochondrial Research Group, Genetics and Genomic Medicine Department UCL Great Ormond Street Institute of Child Health London UK; ^2^ Metabolic Unit Great Ormond Street Hospital for Children NHS Foundation Trust London UK; ^3^ Department of Laboratory Medicine and Pediatrics, Laboratory Genetic Metabolic Diseases, Emma Children's Hospital Amsterdam UMC Location University of Amsterdam Amsterdam The Netherlands; ^4^ Amsterdam Gastroenterology Endocrinology Metabolism, Inborn Errors of Metabolism Amsterdam The Netherlands

**Keywords:** cardiolipin, cell trafficking, MAM, MERC, MICOS, mitochondrial lipid biosynthesis, organellar crosstalk, primary mitochondrial disease

## Abstract

Mitochondria are dynamic cellular organelles with complex roles in metabolism and signalling. Primary mitochondrial disorders are a group of approximately 400 monogenic disorders arising from pathogenic genetic variants impacting mitochondrial structure, ultrastructure and/or function. Amongst these disorders, defects of complex lipid biosynthesis, especially of the unique mitochondrial membrane lipid cardiolipin, and membrane biology are an emerging group characterised by clinical heterogeneity, but with recurrent features including cardiomyopathy, encephalopathy, neurodegeneration, neuropathy and 3‐methylglutaconic aciduria. This review discusses lipid synthesis in the mitochondrial membrane, the mitochondrial contact site and cristae organising system (MICOS), mitochondrial dynamics and trafficking, and the disorders associated with defects of each of these processes. We highlight overlapping functions of proteins involved in lipid biosynthesis and protein import into the mitochondria, pointing to an overarching coordination and synchronisation of mitochondrial functions. This review also focuses on membrane interactions between mitochondria and other organelles, namely the endoplasmic reticulum, peroxisomes, lysosomes and lipid droplets. We signpost disorders of these membrane interactions that may explain the observation of secondary mitochondrial dysfunction in heterogeneous pathological processes. Disruption of these organellar interactions ultimately impairs cellular homeostasis and organismal health, highlighting the central role of mitochondria in human health and disease.

## 1 INTRODUCTION

The complexity of the interactions among cellular organelles is fascinating and far from being fully understood. Nevertheless, the importance of these crosstalk and networking systems for cellular functioning and survival and overall organismal wellbeing is becoming apparent. The knowledge of human diseases associated with structural proteins and enzymes involved in inter‐organellar crosstalk is constantly growing.

Mitochondria are highly dynamic subcellular organelles with a multitude of functions including bioenergetics, anaplerosis, biosynthesis of complex molecules and key roles in cellular signalling, for example in apoptotic and immune pathways.^1^ Understanding the structure, function and dynamics of the mitochondrial membranes, and the complex processes required for synthesis and maintenance of their lipid components, is an emerging area of mitochondrial medicine.^2^

Primary mitochondrial diseases (PMD) have been defined as disorders caused by pathogenic genetic variants that lead primarily or secondarily to dysfunction of oxidative phosphorylation (OXPHOS) or other disturbances of mitochondrial structure and function.^3^ These disorders are defined by their clinical complexity, notoriously presenting with any symptom affecting any organ or combination of organs at any age,^4^ which in turn leads to diagnostic odysseys for affected patients.^5^

In this review we aim to provide an overview of the complexity of mitochondrial membrane structure and function and crosstalk with other cellular organelles. We will discuss complex lipid biosynthesis for the mitochondrial membranes, in particular the structure and function of the highly specialised mitochondrial lipid cardiolipin, and how abnormalities of mitochondrial membranes and their lipid components contribute to human disease. This is a rapidly expanding field, and we foresee that several new diseases will be found associated with these pathways. Finally, we will consider mitochondrial membrane dynamics and interactions with other cellular organelles at membrane contact sites and how these processes contribute to inter‐organellar crosstalk, which is increasingly recognised to be critical for cellular health.

## 2 METHODS

A literature review was performed in the Pubmed database until 15 February 2024 searching for *gene/protein* AND mitochondria or mitochondria AND *single organelles* (e.g., endoplasmic reticulum (ER)) or mitochondrial disease AND *specific pathway* (e.g. membrane biosynthesis). Some papers were also identified as secondary sources cited by publications identified in the literature search.

## 3 INSIDE THE MITOCHONDRIA

### 3.1 Mitochondrial membranes and compartments

**FIGURE 1:**
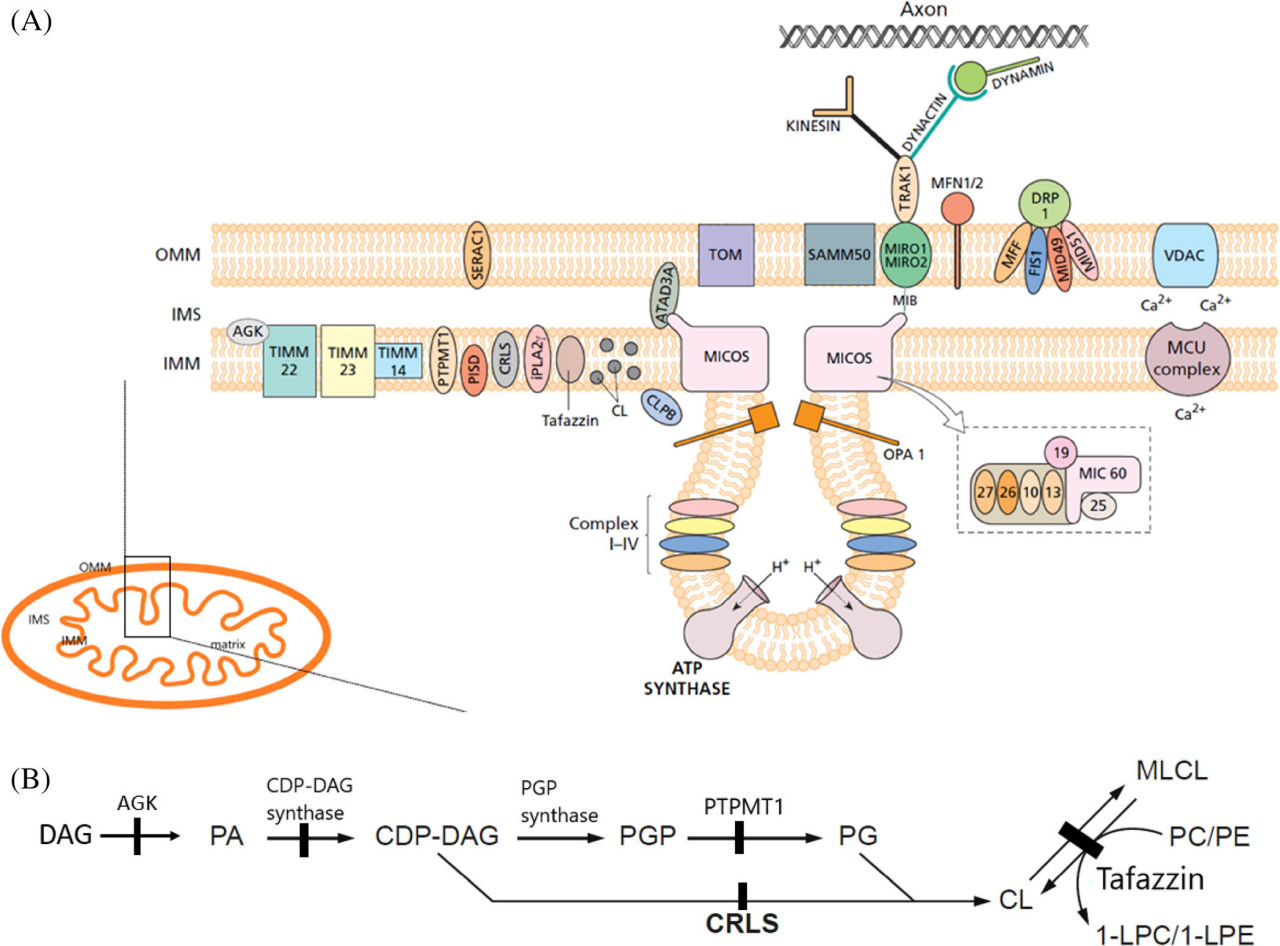
(A) Mitochondrial membrane structure with localisation of enzymes involved in cardiolipin biosynthesis, MICOS complex and mitochondrial cristae structure, and components involved in mitochondrial dynamics and trafficking and their interactions. AGK, acylglycerol kinase; CL, cardiolipin; CRLS, cardiolipin synthase; DRP1, dynamin‐related protein 1; FIS1, mitochondrial fission protein 1; IMM, Inner Mitochondrial Membrane; IMS, Inter Membrane Space; iPLA2γ, calcium‐independent phospholipase A2; MCU, mitochondrial calcium uniporter; MFF, mitochondrial fission factor; MFN1/2, mitofusin 1/2; MIB, mitochondrial intermembrane space bridging complex; MICOS, mitochondrial contact site and cristae organizing system; MiD49/51, mitochondrial dynamics protein of 49/51 kDa; OMM, Outer Mitochondrial Membrane; OPA1, Optic Atrophy Type 1; PISD, phosphatidylserine decarboxylase; PTPMT1, protein tyrosine phosphatase mitochondrion 1; SAMM50, sorting and assembly machinery 50 kDa; TIMM14 translocase of IMM 14; TIMM22, translocase of IMM 22; TIMM23 translocase of IMM 23; TOM, translocase of the outer mitochondrial membrane; TRAK, trafficking of kinesin‐binding; VDAC, voltage‐dependent anion‐selective channel. (B) Details of cardiolipin biosynthesis pathway. The black markers identify enzymatic defects leading to disease. AGK, acylglycerol kinase encoded by *AGK*; CDP‐DAG, cytidine diphosphate‐diacylglycerol encoded by *TAMM41*; CL, cardiolipin; CRLS, cardiolipin synthase encoded by *CRLS1*; DAG, diacylglycerol; MLCL, monolysocardiolipin; PA, phosphatidic acid; PGP, phosphatidylglycerolphosphate; PTPMT1, protein tyrosine phosphatase mitochondrion 1 encoded by *PTPMT1*; PG, phosphatidylglycerol; PC/PE, phosphatidylcholine/phosphatidylethanolamine; 1‐LPC/1‐LPE, 1‐lysophosphatidylcholine/1‐lysophosphatidylethanolamine.

Traditionally, the mitochondrion has been thought of as having four compartments: the outer mitochondrial membrane (OMM), the inner mitochondrial membrane (IMM), the intermembrane space (IMS) and the matrix (Figure 1A, bottom left). Recent advances in super resolution microscopy have allowed greater understanding of the invaginations of the IMM, known as cristae, and have revealed further mitochondrial sub‐compartments.^6^ The cristae junctions connect the cristae to the IMM, which can now be seen to have two surfaces: the inner boundary membrane (IBM) that runs parallel to the OMM, and the deeply involuted cristae membrane. Cristae have a different architecture in different cell types. For example, they are densely packed in cardiac mitochondria, but sparser and thinner in hepatic mitochondria, which may reflect the different bioenergetic states and functions of these organs.^7^ Remarkably, different cristae within the same mitochondrion can have different membrane potentials, allowing us to envisage how fine‐tuning of bioenergetics and distribution of metabolites may occur at a submitochondrial level.^8^

### 3.2 Clinical recognition of disorders of the mitochondrial membranes, lipid biosynthesis and cristae remodelling

Within the context of the complexity and heterogeneity of PMDs, there appear to be some recurring themes in diseases of mitochondrial lipid biosynthesis, remodelling and proteostasis, mitochondrial contact site and cristae organizing system (MICOS) assembly and mitochondrial dynamics. These include cardiomyopathy, cataracts, optic neuropathy, peripheral neuropathy, neurodegeneration, hyperammonaemia and 3‐methylglutaconic aciduria (3MGA), although none of these is a universal feature of this group of disorders.^9^

### 3.3 Mitochondrial membrane biosynthesis

Biosynthesis of complex lipids and associated disorders is an expanding field of metabolic medicine.^10^ Lipid biosynthesis mostly occurs in the ER, but also occurs in peroxisomes and mitochondria. The importance of mitochondrial lipid biosynthesis is highlighted by the existence of a specialised lipid, cardiolipin, that is unique to the IMM. Cardiolipin confers highly specialised functions on the IMM, including the ability to maintain an electrochemical gradient across its two surfaces to sustain OXPHOS, allow formation of cristae by its unique molecular properties and to import and sort more than 1000 proteins synthesised on cytosolic ribosomes into their correct mitochondrial compartments.^11^, ^12^

The protein component of the OMM and IMM is critical not only for its structural function but also for its role in forming functional protein import complexes, regulating mitochondrial dynamics, and supercomplex assembly and function^13^ (Figure 1A).

The IMM contains a combination of phospholipids that are imported from the ER and adjacent organelles and other lipids that are synthesised within the mitochondria from imported precursors. Lipids synthesised within the mitochondrion include cardiolipin, phosphatidylethanolamine (PE) and phosphatidylglycerol (PG). Hence, both intramitochondrial lipid biosynthesis and lipid transport to the OMM and IMM are essential for intact and functional mitochondrial membranes.^14^

Cardiolipin synthesis begins with the transport of phosphatidic acid (PA) to the IMM via the transport of the inner membrane (TIM) protein import system (Figure 1A). Components of this complex are the Translocase of IMM 22 (TIMM22), TIMM14 and TIMM23. Mitochondrial lipid biosynthesis enzymes also play a structural role in these protein import complexes, including acylglycerol kinase (AGK) that catalyses the synthesis of lysophosphatidic acid (LPA) and phosphatidic acid (PA) from monoacylglycerol and diacylglycerol, respectively, in the OMM,^15^, ^16^, ^17^, ^18^ and is a component of the TIM22 complex (Figure 1A). TAMM41 encodes the mitochondrial cytidine diphosphate‐diacylglycerol (CDP‐DAG) synthase that catalyses the conversion of PA into CDP‐DAG.^19^ In an interesting parallel with AGK, TAMM41 is also required for the integrity and activity of the TIMM23 translocase, another component of the mitochondrial protein import apparatus. These synergies hint at coordination between mitochondrial lipid biosynthesis and mitochondrial protein import, suggesting that organelle biogenesis is a tightly regulated process, titrating synthesis and import of all lipid and protein components necessary for mitochondrial structure and function.^19^

The committed step in the cardiolipin synthesis pathway is the transfer of the activated phosphatidyl group from CDP‐DAG to the *sn‐1* position of glycerol‐3‐phosphate by phosphatidyl glycerol phosphate (PGP) synthase to yield PGP. In the following step of the pathway, the newly formed PGP is hydrolyzed to PG by protein tyrosine phosphatase mitochondrion 1 (PTPMT1),^20^ recently reported as disease causing by Falabella et al. (oral communication, EuroMit 2023). In the final step of the pathway, PG receives an activated phosphatidyl group from another CDP‐DAG molecule to form cardiolipin. This reaction is catalyzed by the enzyme cardiolipin synthase (encoded by *CRLS1*) localized exclusively in the IMM (Figure 1A,B).

The calcium‐independent phospholipase A2 (iPLA2γ) encoded by the *PNPLA8* gene is a phospholipase active on the *sn‐1* and *sn‐2* positions of phospholipids. This enzyme, belonging to the patatin‐like protein family is localized to the IMM but also present in peroxisomes, and possibly the ER (Figure 1A). It is implicated in phospholipid remodelling, particularly of plasmalogens and fatty acids such as arachidonic acid, docosahexaenoic acid and linoleic acid. iPLA2γ is activated by cardiolipin when processing arachidonic acid‐containing phosphatidylcholines and can deacylate oxidized cardiolipin to form monolysocardiolipin (MLCL), suggesting a role in the regulation of oxidative stress,^21^ lipid second messenger generation, eicosanoid signalling and cardiolipin remodelling.^20^, ^22^, ^23^ Finally, the phospholipid‐lysophospholipid transacylase tafazzin remodels cardiolipin, via MLCL, into its mature form by adding tissue specific acyl chains necessary for optimal mitochondrial function.^20^

Lysophosphatidylglycerol (LPG) acyltransferase 1 (LPGAT1) is an acyltransferase that catalyses PG remodelling at Mitochondria‐Associated Membranes (MAM) with LPG and acyl‐CoA as substrates. LPGAT1 has a role in regulating mitochondrial PG transport by specifically interacting with the prohibitin/TIM complex, an interaction that allows coupling of PG remodelling at the MAM with mitochondrial import of newly remodelled PG^24^ (Figure 1A). Deletion of *lpgat1* in mouse embryonic fibroblasts prevents mitochondrial transport of newly remodelled PG, resulting in a loss of mitochondrial cristae structure and respiration, associated with cardiomyopathy, hearing loss, liver impairment and 3MGA in a mouse model,^25^ suggesting that cardiolipin may be affected by lack of PG. No human *LPGAT1* related disease has been described to date, but the phenotype of the mouse model partially recapitulates the human 3MGA, deafness, and encephalopathy, Leigh‐like (MEDGEL) syndrome caused by deficiency of SERAC1, another PG remodeller also localised to the MAM.^24^, ^26^ It is possible that human LPGAT1 deficiency may be found to be another cause of MEGDEL in the future. PG remodelling by SERAC1 is essential for mitochondrial functions and intracellular cholesterol trafficking. A new role for SERAC1 has been suggested recently, namely stabilising the mitochondrial serine importer sideroflexin 1 (SFXN1). *SERAC1* knockdown in HEK293 cells, patient cells and a mouse model impaired serine import from the cytosol and disrupted the one carbon cycle, leading to reduced nucleotide availability and mtDNA depletion.^27^

Another mitochondrially synthesised phospholipid, PE, which plays a structural and functional role for the mitochondrial cristae, is synthesised from phosphatidylserine (PS) by phosphatidylserine decarboxylase (PISD), located in the IMM^28^, ^29^ (Figure 1A). PISD is also reported to support autophagy, particularly by playing a role in autophagosome–lysosome fusion through PE synthesis^28^, ^30^ and is involved in the mechanism of fibrogenesis by regulating phospholipid metabolism.^31^

### 3.4 Disorders of mitochondrial membrane lipid biosynthesis

**Acylglycerol kinase deficiency** (*AGK*, biallelic variants, autosomal recessive inheritance, MIM #610345), first described by Sengers in 1975, is a rare mitochondrial disease (prevalence < 1:1 000 000^16^). Forty‐three cases have been described in the literature to date.^15^ The main clinical features are cataracts, hypertrophic cardiomyopathy and muscle weakness with increased plasma lactate level.^15^, ^32^, ^33^ Typically, two phenotypic forms are described, a severe infantile form, which can present as early as the first hours of life and a milder one with onset after the neonatal period.^15^ Cataract is the most common feature and was reported to be an isolated finding in three cases.^34^ Cardiomyopathy, usually hypertrophic but sometimes dilated, with variable left ventricular non‐compaction,^16^ is thought to be responsible for the morbidity of this disease, which is usually due to heart failure. High lactate levels have been associated with a poorer prognosis.^15^ Other biochemical findings are abnormal urinary organic acids with 3MGA.^15^ OXPHOS studies in muscle biopsy and fibroblasts revealed isolated respiratory enzyme deficiencies in some cases, or a combined deficiency of complex I and V in other cases.^15^, ^17^ Homozygous missense variants appear to be associated with a more severe phenotype and infantile death, while compound heterozygous and splicing variants seem to lead to a less severe presentation.^15^, ^16^, ^17^

**Dilated cardiomyopathy with ataxia** (*DNAJC19*, biallelic variants, autosomal recessive inheritance, MIM #212350) (DCMA) sometimes known as 3MGA type V, is another autosomal recessive mitochondrial cardiomyopathy. This disorder has been reported in 18 Canadian Dariusleut Hutterite individuals and two Finnish brothers. Dilated cardiomyopathy and left ventricular non‐compaction, with onset in infancy or early childhood, was variably associated with long QT interval. Other clinical features included microcytic anaemia, growth retardation, mild ataxia, mild muscle weakness, genital anomalies in males, optic atrophy and delayed psychomotor development. All affected individuals had a homozygous pathogenic variant in *DNAJC19* encoding the TIMM14 component of the IMM protein import system, thus showing aetiological similarities to Sengers syndrome.^18^ Cardiac failure was a frequent cause of death.^35^, ^36^

**TAMM41 deficiency** (*TAMM41*, biallelic variants, autosomal recessive inheritance, MIM #620139) is one of two new disorders of cardiolipin synthesis reported in 2022. *TAMM41* encodes CDP‐DAG synthase. Three unrelated patients presenting with lethargy at birth associated with hypotonia, developmental delay, myopathy and ptosis were found to have compound heterozygous variants in *TAMM41*.^37^ Combined deficiency of respiratory chain complexes I and IV was noted in two of the three cases. An unusual observation for this group of disorders, however, was that none of the affected individuals with TAMM41 deficiency had cardiomyopathy, and no reported increase in plasma lactate levels.^37^ The reasons for this difference are not known, but it is possible that *TAMM41* defects will be identified in the future in individuals with mitochondrial cardiomyopathy.

**Cardiolipin synthase deficiency** (*CRLS1*, biallelic variants, autosomal recessive inheritance, MIM #620167) is the second disorder of cardiolipin synthesis reported in 2022. It is caused by biallelic pathogenic variants in *CRLS1*. Four patients, including two siblings, were reported to have progressive infantile onset mitochondrial encephalopathy with bull's eye maculopathy (an unusual feature in PMD), auditory neuropathy, diabetes insipidus, autonomic instability and hypertrophic cardiomyopathy leading to early death.^38^

**PNPLA8 deficiency** (*PNPLA8*, biallelic variants, autosomal recessive inheritance, MIM #618889). A total of nine patients have been reported with pathogenic variants in *PNPLA8* which encodes phospholipase iPLA2γ which is involved in cardiolipin remodelling in the IMM.^22^, ^23^ The clinical phenotype of PNPLA8 deficiency ranges from congenital microcephaly, epileptic encephalopathy, lactic acidosis and early death at the most severe end, through a childhood neurodegenerative presentation to adulthood onset cerebellar ataxia and peripheral neuropathy.^39^, ^40^, ^41^ Brain abnormalities are reported to be present in most cases, with basal ganglia and corpus callosum changes.^23^, ^40^, ^41^

**Tafazzin deficiency** (*TAFAZZIN*, hemizygous variants, X‐linked inheritance, MIM #614725), Barth syndrome, is an X‐linked disorder caused by a deficiency of tafazzin, an acyltransferase that catalyses the remodelling of cardiolipin in mitochondrial membranes. Tafazzin deficiency leads to abnormal cardiolipin acylation, with inadequate formation of mature cardiolipin—mostly tetralinoleyl‐cardiolipin—and accumulation of monolysocardiolipin (MLCL).^42^ The disorder is defined clinically by dilated cardiomyopathy and skeletal myopathy, variably associated with (cyclical) neutropaenia and growth disturbance. Affected boys are said to have a characteristic facial appearance, with a tall broad forehead, round face with full cheeks, prominent ears and deep‐set eyes.^43^ These facial features are less evident after puberty and the small boys usually exhibit catch‐up growth, frequently resulting in tall adults.^43^ The presence of cardiomyopathy associated with skeletal myopathy and neutropaenia in a boy should prompt a series of investigations. Cardiomyopathy may not always be present at diagnosis^44^ and neutropaenia can be intermittent.^43^ Urine organic acid analysis might reveal the presence of 3MGA but this is not a specific finding for Barth syndrome and its absence should not rule out the diagnosis. In Barth syndrome mature cardiolipin level is decreased, while increased levels of MLCL and altered cardiolipin acyl composition occur, leading to a pathognomonic increase of the MLCL/cardiolipin ratio.^45^, ^46^

**SERAC1 deficiency** (*SERAC1*, biallelic variants, autosomal recessive inheritance, MIM #614739), first described in 2006,^47^ is a rare mitochondrial disorder (prevalence 0.09: 100 000^48^) which presents with 3MGA with sensorineural hearing loss, variable hepatopathy, encephalopathy and Leigh syndrome spectrum.^49^, ^50^ In the infantile presentation, neonatal hypoglycaemia and sepsis‐like episodes can be the first disease signs. It progresses to severe hypotonia, motor regression, dystonia and spasticity.^49^ MEGDEL syndrome may also manifest later in childhood with a milder, oligosymptomatic phenotype.^50^ To date a least 102 cases have been reported in the literature. The main biochemical findings are increased plasma lactate, reduced cholesterol and 3MGA. 3MGA levels do not correlate with the severity of the presentation, and there is no obvious genotype–phenotype correlation.^49^, ^50^

**Phosphatidylserine decarboxylase deficiency** (*PISD*, biallelic variants, autosomal recessive inheritance, MIM #612770) is an extremely unusual PMD, with a primary manifestation of spondyloepimetaphyseal dysplasia, variably accompanied by a constellation of other symptoms including microcephaly, early‐onset retinal degeneration, cataract, hearing loss, intellectual disability, severe joint laxity and short stature, eponymously named Liberfarb syndrome.^29^, ^51^, ^52^

### 3.5 3‐Methylglutaconic aciduria

Although 3MGA is not a specific finding for primary mitochondrial diseases, it is overrepresented in urine samples from patients with suspected mitochondrial disease compared to samples from patients with other inherited metabolic diseases.^53^ Some conditions are associated with marked 3MGA as a biochemical hallmark, notably deficiency of 3‐methylglutaconyl‐CoA hydratase.^54^ This enzyme, also known as AU‐binding homolog of enoyl‐coenzyme A (AUH), catalyses the last step of leucine degradation. AUH deficiency came to be known as 3MGA type I, with other disorders associated with 3MGA classified as 3MGA type II (Barth syndrome), 3MGA type III (defects in OPA3) and 3MGA type IV historically referring to miscellaneous mitochondrial disorders not genetically defined.^55^, ^56^ Later, a simplified classification proposed that AUH deficiency should be referred to as primary 3MGA, with all other causes representing secondary 3MGA.^57^

With the identification of deficiency of the CLPB mitochondrial disaggregase as a novel cause of 3MGA, we hypothesised that this metabolite is a biomarker of mitochondrial membrane defects.^9^ The discovery by an Australian group that AUH is a dual function enzyme, required for normal IMM structure and maintenance of a mitochondrial reticular network in addition to its role in leucine catabolism,^58^ has led us to modify our hypothesis. We now suggest that the 3‐methylglutaconyl‐CoA hydratase function of AUH is critically intertwined with its role in maintaining IMM health. We propose a common mechanism for all conditions with a distinctive 3MGA, namely the impairment of 3‐methylglutaconyl‐CoA hydratase activity. The location of AUH in the IMM make this enzyme susceptible to reduced function when mitochondrial membrane disruption occurs, as is the case with defects of enzymes involved in mitochondrial complex lipid biosynthesis and remodelling and protein quality control, leading to 3MGA as a unifying feature of these disorders (Table 1).

**TABLE 1:** Summary table of diseases associated with mitochondrial membrane biosynthesis, MICOS complex and cristae remodelling, mitochondrial dynamics and interorganellar network.

Gene	Phenotype	3MGA	MIM number	Inheritance
Mitochondrial membrane biogenesis and remodelling				
*SERAC1*	3MGA with sensorineural deafness, variable hepatopathy, encephalopathy, Leigh syndrome spectrum	+	614739	AR
*CRLS1*	Progressive infantile onset mitochondrial encephalopathy, bull's eye maculopathy, auditory neuropathy, diabetes insipidus, autonomic instability, hypertrophic cardiomyopathy		620167	AR
*PNPLA8*	Congenital microcephaly, epileptic encephalopathy, lactic acidosis and early death/childhood neurodegenerative presentation/adulthood onset cerebellar ataxia and peripheral neuropathy		251950	AR
*TAFAZZIN*	Barth syndrome (dilated cardiomyopathy, neutropaenia)	+	302060	XL
*PISD*	Liberfarb syndrome		618889	AR
*MICOS13* (*QIL1, C19ORF70*)	Early onset hepatopathy, encephalopathy, cerebellar atrophy	+	618329	AR
*APOO*	Myopathy, intellectual disability		N/A	XL
*CHCHD10*	MtDNA instability disorder/frontotemporal dementia‐amyotrophic lateral sclerosis clinical spectrum/late‐onset spinal motor neuropathy and CMT type 2		61209 615911 615048	AD
*CHCHD2*	Parkinson disease		616710	AD
*SLC25A46*	Leigh syndrome, optic atrophy, severe axonal sensorimotor neuropathy, lethal ponto‐cerebellar atrophy		616505 619303	AR
Mitochondrial protein import				
*AGK*	Cataract, hypertrophic cardiomyopathy, myopathy	+	212350	AR
*TAMM41*	Hypotonia, developmental delay, myopathy, ptosis	+	620139	AR
*DNAJC19*	Dilated or non‐compaction cardiomyopathy, with onset in infancy or early childhood, variably associated with long QT interval. Microcytic anaemia, growth retardation, ataxia, hypotonia, optic atrophy, developmental delay	+	610198	AR
*TIMM22*	Hypotonia, raised lactate	+	607251	AR
*TIMM8A*	Mohr–Tranebjaerg syndrome		300356	XL
*TIMM50*	3MGA, early‐onset seizures, severe psychomotor delay, intellectual disability	+	607381	AR
*PAM16*	Spondylometaphyseal dysplasia		614336	AR
Mitochondrial protein quality control				
*TMEM70*	Neonatal encephalopathy, cardiomyopathy, lactic acidosis, hyperammonaemia	+	614052	AR
(*CLPB SKD3*)	3MGA, severe congenital neutropaenia, cataracts, ovarian insufficiency	+	616271, 619835, 61981	AR, AD
*ATAD3A*	Harel–Yoon syndrome	+	617183	AD, AR
	Pontocerebellar hypoplasia, hypotonia, and respiratory insufficiency syndrome		618810	AR
*CLPP*	Perrault syndrome		601119	AR
*LONP1*	CODAS syndrome		605490	AR
*OPA3*	3MGA	+	258501	AR
	Optic atrophy with cataract		165300	AD
*AFG3L2*	Optic atrophy		618977	AD
	Spinocerebellar ataxia		610246	AD
	Spastic ataxia		614487	AR
*SPG7*	Spastic paraplegia		607259	AD, AR
*HTRA2*	3MGA, hypotonia, neutropaenia, seizures, developmental delay, intellectual disability	+	617248	AR
Mitochondrial dynamics and interorganellar interactions				
*TRAK1*	Developmental delay and epileptic encephalopathy		608112	AR
*MFN2*	CMT2A2A		609260	AD
	CMT2A2B		617087	AR
	Hereditary motor and sensory neuropathy VIA		601152	AD
	Multiple symmetric lipomatosis		151800	AR
*OPA1*	Optic atrophy		605290	AD
*DNM1L*	Epileptic encephalopathy, microcephaly, DD and pain insensitivity; Optic atrophy		614388, 610708	AR, AD AD
*MSTO1*	Mitochondrial myopathy and ataxia		617675	AD, AR
*MIEF2*	Muscle weakness, exercise intolerance		615498	AR
*DNM2*	CMT2M		606482	AD
*MFF*	Encephalopathy		614785	AR
*SPG20*	Troyer syndrome		607111	AR
*SPATA5*	Neurodevelopmental disorder with hearing loss, seizures, and brain abnormalities		613940	AR
*STAT2*	Immune deficiency		600556	AR
*VPS13D*	Spinocerebellar ataxia		608877	AR
*GDAP1*	CMT2K		607831	AD, AR
*RAB7*	CMT2B		600882	AD
*MCOLN1*	Mucolipidosis type IV		252650	AR
*MCU1*	Proximal myopathy with extrapyramidal signs		605084	AR

*Note*: See text for detailed description and pathophysiology.

Abbreviations: 3MGA, 3‐methylgutaconic aciduria; AD, autosomal dominant; AR, autosomal recessive; CMT, Charcot–Marie–Tooth; mtDNA, mitochondrial DNA; XL, X‐linked.

### 3.6 Treatment approach for disorders of the mitochondrial membranes

Unfortunately, no curative treatment is available for these conditions, which are mainly managed with a symptomatic multidisciplinary approach.^15^, ^16^, ^17^, ^49^, ^50^ Some specific therapeutic approaches have been proposed for this group of diseases, although some of these are still at a preclinical stage and have not progressed to the clinic. For PISD deficiency, several studies have suggested that ethanolamine supplementation to feed the ER PE synthesis pathway could provide enough PE to the mitochondria to support normal function and morphology. However, more investigations into ethanolamine supplementation in cellular and animal models are warranted.^28^, ^52^, ^59^ Elamipretide is a tetrapeptide that targets cardiolipin in the IMM with some evidence of improvement of mitochondrial function particularly in cardiomyopathies.^60^ Ongoing clinical trials are evaluating its safety and efficacy in patients with Barth syndrome (ClinicalTrials.gov ID NCT03098797) but given its function it is possible that it could be beneficial in other mitochondrial membrane biosynthesis defects. AAV9 gene therapy has been attempted in a mouse model of tafazzin deficiency and led to restored mitochondrial and cytoskeleton structure and prevention but not full reversal of cardiomyopathy.^61^, ^62^ Enzyme replacement therapy with recombinant human tafazzin is under investigation.^63^

### 3.7 Cristae remodelling and cristae organizing system MICOS complex

As mentioned above, a highly specialised function of the IMM is its invagination into numerous cristae which allows compartmentalisation of multiple mitochondrial functions, including mtDNA replication and maintenance, mitochondrial protein synthesis and bioenergetics. Abnormal cristae morphology visualised by electron microscopy has long been recognised as an ultrastructural hallmark of PMD.^64^

A major advance in recent years has been the recognition of highly specialised proteins at the cristae junction that form the MICOS complex^65^ (Figure 1A). Subunits of the MICOS complex have been implicated in IMM shaping, mitochondrial import, mtDNA maintenance, nucleoid formation and morphology, maintenance of the mitochondrial membrane potential, lipid transport, stability of OXPHOS complexes and mitophagy. The MICOS complex acts as a stable physical anchor facilitating mitochondrial movement in cells.^6^, ^66^, ^67^ Moreover, the MICOS complex seems to be more than just a structural component and appears to play key roles in linking the IMM with cytosolic reactions.^6^

MICOS proteins were first characterised in yeast and more recently their human homologues have been identified and defects of these proteins have been linked to human disease.^13^, ^68^ MICOS is composed of several subunits, not all fully recognised,^68^ located on the IMM and in the IMS. The MICOS subunits are organized into two subcomplexes: MIC60 subcomplex (MIC60–MIC19–MIC25) and MIC10 subcomplex (MIC13–MIC10–MIC26–MIC27), bridged together by MIC13.^6^, ^69^, ^70^ Deletion of MICOS proteins in yeast led to loss of cristae junctions.^66^, ^71^ MIC60 and MIC27 have been shown to bind to cardiolipin.^72^, ^73^ MIC10 and MIC60 appear to bend the IMM to induce negative curvature,^74^ whilst dimers and oligomers of the ATP synthase contribute to positive curvature at cristae tips.^75^ MIC60 is an integral IMM protein and a major component of the MICOS complex. It is reported to be a substrate of PINK1, a well described Parkinson disease‐associated gene, and to be important for maintaining cristae junctions. MIC60 also appears to be a controller of mitochondria‐dependent apoptotic cell death, suggesting a potential involvement of this subcomplex in Parkinson disease pathogenesis.^76^

Cristae formation and remodelling is regulated by multiple proteins including the MICOS complex, ATP synthase dimers and the mitochondrial fusion protein optic atrophy 1 (OPA1). The OMA1 protease that regulates the activity of OPA1^77^ has also been linked to MICOS complex assembly, cristae morphology and apoptosis.^78^ Solute carrier family 25 member 46 (SLC25A46) is an integral protein of the OMM and interacts with mitofilin, OPA1, mitofusin 2 (MFN2), and the MICOS complex to maintain mitochondrial cristae structure and dynamics. It also plays a role in lipid homeostasis and phospholipid transfer from the ER.^77^

Other protein components of the mitochondrial membrane have been linked to the MICOS complex. *CHCHD2* and *CHCHD10* encode two homologous proteins that belong to the mitochondrial coiled helix coiled helix (CHCH) domain protein family.^79^ *CHCHD10* and *CHCHD2* have been linked to autosomal dominant neurological diseases associated with MICOS complex disassembly but have not yet been formally demonstrated to be part of the MICOS complex. Biochemically, CHCHD10 defects result in decreased MICOS complex organization, together with reduced copy number and instability of mtDNA.^80^ *CHCHD2* gene expression correlates closely with OXPHOS genes suggesting a modulatory role for CHCHD2 and CHCHD10 in highly energetic tissues. Loss of CHCHD2 also disrupts the morphology of mitochondrial cristae, so this protein is hypothesised to play a role in inhibiting mitochondrial apoptosis and regulating mitophagy.^81^

SAMM50 is a component of the sorting and assembly machinery (SAM) and contributes to cristae stability by interacting with core proteins of the MICOS complex.^82^ It is also predicted to play a role in regulating PINK1‐parkin mediated mitophagy.^82^ The MICOS complex also functions as an anchor for mitochondrial transport and together with SAM represents the mitochondrial intermembrane space bridging (MIB) complex.^6^ MICU1, a key regulator of the mitochondrial calcium uniporter MCU, has also been shown to interact with MICOS proteins (MIC60 and MIC19) and appears to regulate cristae structure independently of its role in mitochondrial calcium import.^83^, ^84^

Metabolic factors can also impact cristae morphology, including glucose and ADP levels, starvation, hypoxia and apoptosis.^66^ Accumulation of Krebs cycle intermediates is a well‐recognised feature of PMDs, presumably because loss of reducing equivalents leads to stalling of the Krebs cycle. A recent link between the Krebs cycle and cristae structure is the observation that α‐ketoglutarate accumulation leads to D‐2‐hydroxyglutarate accumulation, which in turn leads to accumulation of 3‐hydroxypropionate, and that the latter can inhibit the action of MIC60 in membrane shaping.^85^

### 3.8 Disorders of the MICOS complex

Pathogenic variants in MICOS proteins have been linked to heterogeneous human diseases, ranging from early onset neurodevelopmental disorders to late onset myopathies, Parkinson disease and amyotrophic lateral sclerosis.

**MICOS13 deficiency** (*MICOS13*, also known as *QIL1*, *C19ORF70*, biallelic variants, autosomal recessive inheritance, MIM #616658). *MICOS13* mutation leads to early onset fatal liver disease and encephalopathy with cerebellar atrophy. Hyperlactatemia and 3MGA has been reported.^86^, ^87^, ^88^ Loss of MIC13 causes subsequent depletion of subunits of the MIC10 subcomplex with the MIC60 subcomplex remaining intact.^69^ Interestingly, one clinical case was described with low complex I, III and IV activities and decreased mtDNA copy number,^86^ supporting the hypothesis that MICOS13 might be involved in mtDNA replication, in common with other MICOS components.^80^ Based on this some authors suggest that *MICOS13* should be classified as a causative gene of mtDNA depletion syndrome.^86^

**MICOS complex subunit MIC26 deficiency** (*APOO*, X‐linked inheritance, MIM #300753) has been reported in two case reports, variably associated with mitochondrial myopathy with lactic acidosis, cognitive impairment and autistic features, or with a lethal mitochondrial disease with a progeria‐like phenotype associated with partial agenesis of the corpus callosum, bilateral congenital cataracts, hypothyroidism and severe immune deficiencies.^89^, ^90^ Studies of respiratory chain enzymes showed no deficiencies nor altered respiration.^89^

**CHCHD10 deficiency** (*CHCHD10*, monoallelic variants, autosomal dominant inheritance, MIM #615903). CHCHD10‐related diseases include mtDNA instability disorder, frontotemporal dementia‐amyotrophic lateral sclerosis (FTD‐ALS) clinical spectrum, late‐onset spinal motor neuropathy (SMAJ) and Charcot–Marie–Tooth disease type 2 (CMT2).^91^ The neurodegenerative phenotype of these diseases seems to be related to a gain of function mechanism with toxicity secondary to protein misfolding.^92^, ^93^

**CHCHD2 deficiency** (*CHCHD2*, monoallelic variants, autosomal dominant inheritance, MIM #616244). *CHCHD2* was the first mitochondrial gene reported to cause Parkinson disease,^94^ but mutations have also been described in Alzheimer disease and in frontotemporal dementia. As with CHCHD10 defects, a gain of function mechanism with misfolded protein toxicity has been suggested as the pathogenic mechanism of neurodegeneration.^93^

**SLC25A46 deficiency** (SLC25A46, biallelic variants, autosomal recessive inheritance, MIM #610826), has been reported to cause Leigh syndrome, optic atrophy spectrum disorder (variably associated with Parkinson disease), severe axonal sensorimotor neuropathy, cerebellar ataxia and lethal ponto‐cerebellar hypoplasia.^95^, ^96^

**ATAD3A** (*ATAD3*, MIM #612316) is involved in mtDNA maintenance via the cholesterol metabolic pathway.^97^ ATAD3A interacts with the MICOS complex and MICOS complex formation is reduced in *ATAD3A* knockout mice.^98^ This suggests that the maintenance of mtDNA may be regulated through interaction between the MICOS complex and ATAD3A. Oligomerisation of ATAD3 was found to be necessary for nucleoid mobility.^99^, ^100^ *ATAD3A* deficiency has been associated with a neurodevelopmental delay syndrome with truncal hypotonia, spasticity, and peripheral neuropathy inherited in an AD or AR fashion (Harel–Yoon syndrome, MIM #617183), and with an AR lethal ponto‐cerebellar hypoplasia, hypotonia and respiratory insufficiency syndrome (MIM #618810). Interestingly, *ATAD3A*‐associated phenotype has shown an enhanced type I IFN signalling, although the pathomechanisms are yet to be determined.^101^

**Transmembrane protein 70 (TMEM70) deficiency** (*TMEM70*, autosomal recessive, MIM #614052) is the most frequently reported cause of nuclear‐encoded ATP synthase deficiency, resulting in a neonatal encephalocardiomyopathy with lactic acidosis and hyperammonaemia.^102^ TMEM70 functions to shuttle the *c* subunit of ATP synthase from the TIMM complex toward OXA1L, a protein required for insertion of integral membrane proteins into the IMM.^103^

**CLPB deficiency** (*CLPB*, *SKD3*, biallelic variants, 3‐methylglutaconic aciduria, type VIIA, autosomal dominant, 3‐methylglutaconic aciduria, type VIIB, autosomal recessive, Neutropaenia, severe congenital, 9, autosomal dominant, MIM #616254) causes an autosomal recessive or dominant 3MGA, variable neurological disease^104^ and neutropaenia. CLPB is an ATP‐fuelled protein disaggregase with a key role in mitochondrial cristae stability. The presence of 3MGA associated with isolated severe congenital neutropaenia when a different mutation is present in the ATP binding site^105^ suggests that this disease may be a link between membrane biosynthesis defects and cristae remodelling disorders.

### 3.9 Mitochondrial dynamics and motility

Mitochondria are extremely dynamic organelles, and this seems to be a critical aspect of wellbeing at every level: organellar, cellular and individual. The two main processes involved in mitochondrial dynamics are the fusion of two mitochondria and mitochondrial fission, through which new mitochondria are formed. These two processes act as organelle and cellular quality control mechanisms and an imbalance between the two, called dysdynamism, has been reported in neurodegenerative, cardiovascular and respiratory disorders.^106^

### 3.10 Mitochondrial fusion

Mitochondrial fusion enables content mixing, particularly of mtDNA and mitochondrial proteins and metabolites, allows viable mitochondrial segments to recover by fusion with healthy elements,^106^, ^107^ and elongated mitochondria resulting from fusion processes produce ATP more efficiently.^108^

MFN1, MFN2 and OPA1 are GTPases that play a central role in mitochondrial fusion, with MFN1 and MFN2 localised in the OMM and OPA1 localised in the IMM (Figure 1A). MFN1 and 2 appear to have overlapping functions and the two proteins may have a different tissue specificity, with MFN1 prominent in the heart and MFN2 in the brain.^109^ MFN2 has also been related to axonal mitochondrial movement by direct interaction with trafficking protein MIRO2. MFN2 has also been found to localise in the ER suggesting a regulatory function in the mitochondria‐ER tethering fusion process, although the nature of this interaction remains controversial.^109^

OPA1 has several other postulated functions in addition to mitochondrial fusion. For example, OPA1 seems to control cristae remodelling during apoptosis independently of mitochondrial fusion and a loss of OPA1 leads to loss of respiratory chain supercomplexes, suggesting a role in supercomplex formation and stability.

### 3.11 Mitochondrial fission

Mitochondrial fission is a complex process, requiring separation of the OMM and the IMM without releasing mitochondrial content into the cytoplasm. Fission starts at mitochondrial–ER contact sites (MERCs). Symmetrical fission has a replicative role and is coordinated with the cell cycle. Asymmetrical fission leads to the creation of a healthy mitochondrion and a smaller senescent one which is then degraded by mitophagy.

DRP1 is a key component of the fission process. It is usually localised in the cytoplasm and under stress conditions is recruited to the OMM by four adaptors: MFF, FIS1, MID49 and MID51 (Figure 1A). On the OMM it catalyses the last step of the fission process.^106^, ^110^ Other elements are certainly involved in this process. In particular, the actin in the cytoskeleton facilitates recruitment of DRP1 at the MAM. Dynamin protein 2 (DYN2) and BAX interacting factor 1 (BIF1) also help DRP1 in mediating mitochondrial fission. Signal Transducer and Activator of Transcription 2 (STAT2) influences DRP1 phosphorylation playing a modulator role in the fission process.^111^

In another link between mitochondrial membrane lipids and proteins, cardiolipin appears to facilitate the oligomerisation of DRP1 and stimulates its GTPase activity to enhance the constriction of the OMM and IMM.^106^

### 3.12 Mitochondrial trafficking

MIRO1 and MIRO2 are mitochondrial rho GTPases that function to traffic mitochondria within cells. MIROs connect to the MIB, particularly with MIC60 and MIC19, and bridge to trafficking of kinesin‐binding (TRAK) motor proteins to distribute mitochondria within cells^112^ (Figure 1A). In particular, MIRO1 and MIRO2, directly inserted into the OMM, bind the TRAK complex to then bind the KIF5 family kinesins for anterograde transport, which is particularly important for energy delivery to the synaptic space.^108^, ^113^ At the other end of the neuron, MIROs together with the TRAK complex bind the dynein/dynactin complex to mediate the retrograde transport of mitochondria towards the neuronal body.^108^ TRAK1 mainly has an axonal distribution and binds kinesin 1 and the dynein/dynactin complex. TRAK2 interacts with the dynein/dynactin complex and is prominent in dendrites.^108^ In terms of regulation, the mechanism of MIRO‐mediated, calcium‐induced arrest of mitochondrial transport remains unclear.^114^

Myosin19 localised in the mitochondria is predicted to have a role in the fission process by stabilising the MERCs by means of ER‐actin filaments, in an ATP‐dependent process^115^ and plays a structural role in the mitochondrial cristae, so that its depletion leads to altered mitochondrial membrane potential and decreased OXPHOS.^116^ Interestingly, Myosin19 has been shown to bind MIRO1 to facilitate microtubule and actin‐based transport in neurons^108^ (Figure 1A).

### 3.13 Disorders of mitochondrial dynamics and trafficking

**MFN2 deficiency** (*MFN2*, autosomal dominant inheritance, MIM #608507) leads to Charcot–Marie–Tooth 2A neuropathy (CMT2A). MFN2 plays a significant role in mitochondrial movement within the neurons alongside its role in the fusion process. Impaired axonal transport is the presumed pathomechanism of neuropathy in this condition.^117^

**OPA1 deficiency** (*OPA1*, autosomal dominant inheritance, MIM #605290), leads to a Dominant Optic Atrophy phenotype.^117^ Of note, although MFN2 and OPA1 are involved in the fusion process, the phenotype of the two deficiencies is very different, probably related to other roles played by these proteins within the mitochondrion.

**DRP1 deficiency** (*DNM1L*, autosomal dominant or recessive inheritance, MIM #603850). Pathogenic variants in *DNM1L* resulting in dysfunctional or absent DRP1 lead to a severe phenotype with neonatal lethality, epileptic encephalopathy, postnatal microcephaly, developmental delay and pain insensitivity^118^, ^119^ and to a milder phenotype with optic atrophy.^120^

**MID49 deficiency** (*MIEF2*, autosomal recessive inheritance, MIM #615498). One patient who presented in childhood with muscle weakness and exercise intolerance, was reported to have a homozygous pathogenic variant in *MIEF2* encoding MID49, one of the DRP1 adaptors, resulting in impaired fission and combined deficiency of complexes I and IV.^121^

**DNM2 deficiency** (*DNM2*, autosomal dominant inheritance, MIM #602378) leads to CMT2B neuropathy, due to absence of functional Dyn2.^122^

**TRAK1 deficiency** (*TRAK1*, autosomal recessive inheritance, MIM #608112) in humans has been associated with developmental delay and epileptic encephalopathy, whereas TRAK2 has not been correlated with a disease yet.^123^, ^124^

**STAT2 deficiency** (*STAT2*, autosomal recessive inheritance, MIM #600556) has been demonstrated to cause immunodeficiency and sterile meningoencephalitis following childhood vaccinations, associated with hyperfused mitochondria similar to mitochondrial appearances observed in DRP1 deficiency.^111^

## 4 OUTSIDE THE MITOCHONDRIA

**FIGURE 2:**
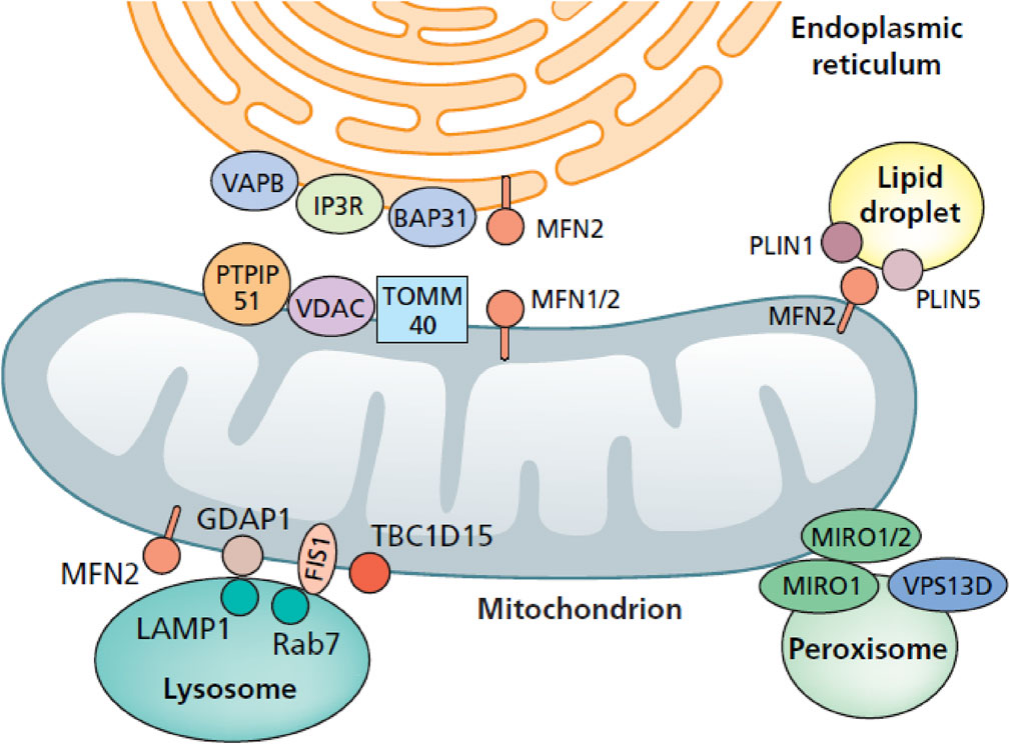
Mitochondrial network and interaction with cellular organelles. BAP31, B‐cell receptor‐associated protein 31; FIS1, mitochondrial fission protein 1; IP3R, inositol triphosphate receptor; MFN2, mitofusin 2; PTPIP51, protein tyrosine phosphatase interacting protein 51; TOMM40, translocase of the OMM 40; TBC1D15, TBC1 Domain Family Member 15; VAPB, vesicle‐associated membrane protein‐associated protein B; VDAC, voltage‐dependent anion‐selective channel; VPS13D, vacuole protein sorting homologue 13D.

In this section, we will discuss interactions between mitochondria and the extra‐mitochondrial milieu, focussing on interactions with the ER, peroxisomes, lipid droplets and lysosomes (Figure 2).

### 4.1 Mitochondria–ER interactions

The interaction between the mitochondria and ER is essential for lipid and calcium exchange and for signalling.^125^ Several proteins of interest localise to the MERCs, the contact sites where the mitochondria and ER interact. On the mitochondrial side there are the voltage‐dependent anion‐selective channel (VDAC), translocase of the OMM 40 (TOMM40) and protein tyrosine phosphatase interacting protein 51 (PTPIP51), while on the ER side there are the vesicle‐associated membrane protein‐associated protein B (VAPB), inositol triphosphate receptors (IP3Rs) and B‐cell receptor‐associated protein 31 (BAP31).^126^ Golgi–ER trafficking of lipids and proteins is mediated by Coat Protein complex I (COPI). COPI depletion in neurons results in neuronal apoptosis.^126^ MFN2 and MIRO1/2 involved in mitochondrial dynamics and transport also seem to localise at the MERCS.^126^, ^127^ MFN2 arguably localises on both the ER and mitochondrial sides, to mediate ER–mitochondrial tethering^109^ (Figure 2).

ER–mitochondrial lipid transfer is essential for mitochondrial membrane biosynthesis. Phospholipids synthesised in the ER are transferred to the mitochondrion to be further metabolised to PE and cardiolipin. MERCs are unsurprisingly enriched with phospholipid biosynthesis enzymes. MIC19, a component of the MICOS complex, appears to regulate MERCs through interaction with SCL25A46 in the OMM and the ER membrane protein complex subunit 2 (EMC2) on the ER side. *MIC19* knockout models show disrupted lipid biosynthesis and mitochondrial formation.^128^

The ER acts as the main cellular reservoir of calcium and the exchange of calcium to the mitochondrion is important for TCA cycle metabolism. Calcium transport is mediated by VDAC and the heat shock protein chaperone GRP75 on the mitochondrial side and IP3R on the ER side. MCU in the IMM, which is a mitochondrial calcium channel, is closely linked to this complex.^125^

### 4.2 Mitochondria–peroxisome interactions

While peroxisomes and mitochondria do not derive from a common ancestor (the origin of mitochondria is endosymbiotic, whereas peroxisomes derive from the endoplasmic reticulum),^129^ several proteins are common to both organelles. In fact, they share not only a few enzymes, but also full metabolic pathways such as β‐oxidation of fatty acids.^130^ Peroxisomes and mitochondria are independent organelles, but their interaction is necessary for optimal cellular function.^114^ The division processes of these two organelles are closely related and use identical factors and enzymes including DRP1, MFF, FIS1 and GDAP1.^114^, ^129^ This suggests efficient crosstalk between peroxisomes and mitochondria. However, the physiological functions of the two organelles are different. In terms of fatty acid metabolism, mitochondria degrade the majority of long‐chain fatty acids to supply acetyl‐CoA for the production of ATP and for anabolic reactions, while peroxisomal β‐oxidation is involved in the degradation of special fatty acids such as very long‐chain and branched‐chain fatty acids as well as the synthesis of bile acids. Thus, the two organelles work together for the metabolism of fatty acids.^130^

The mitochondrial–peroxisome interaction seems to be mediated by MIRO1 and MIRO2 on the OMM, which bind vacuole protein sorting homologue 13D (VPS13D) that also binds the ER, creating a bridge for interaction and lipid exchange.^131^, ^132^ Furthermore, MIRO1 has been reported to localise to the peroxisomal membrane to mediate peroxisomal dynamics^114^ (Figure 2). VPS13D has been demonstrated to be essential for cell and organism survival.^131^

**VPS13D deficiency** (VPS13D, autosomal recessive inheritance, MIM #608877) is associated with severe ataxia, with the phenotype extending to the Leigh syndrome spectrum in one affected individual.^131^

### 4.3 Mitochondria–lipid droplet interactions

Mitochondria are the main producers of energy in the form of ATP to fuel multitudinous cellular functions. In conditions of starvation, it is essential that lipids are delivered to the mitochondria efficiently. Several mechanisms have been hypothesised, including close relationships between mitochondria–ER–peroxisomes,^131^, ^132^, ^133^ but also a direct interaction of mitochondria with lipid droplets.^134^ Lipid droplets are emerging as a new organelle with a single layer surfactant membrane.^135^ In conditions of cell starvation, it has been hypothesised that MFN2 on the mitochondrial side interacts with PLIN family proteins (PLIN1 and PLIN5) on the lipid droplet surface regulating the formation of a contact site^134^ (Figure 2). Also, oxidative stress as a result of reactive oxygen species (ROS) production has been demonstrated to be a trigger to increase lipid droplet formation, and this may be an important mechanism in mitochondrial membrane remodelling.^135^

### 4.4 Mitochondria–lysosome interactions

The interaction between mitochondria and lysosomes is dynamic and bidirectional, with varied purposes, including quality control and transfer of metabolites.^136^ The first relates to mitophagy/autophagy, which is strongly linked to other mitochondrial protein quality control systems such as fusion and fission. Metabolites exchanged between mitochondria and lysosomes include calcium, iron and lipids.^136^, ^137^, ^138^

Mitophagy is a selective autophagy mechanism that acts as a quality control system. In conditions of mitochondrial damage, the mitochondrion targeted for mitophagy is flagged by the presence of microtubule‐associated protein 1A/1B‐light chain 3 (LC3) adapters in both ubiquitin‐dependent and ‐independent pathways to direct it to the lysosomes via autophagosome formation. The ubiquitin‐dependent pathway relies on PINK1 accumulation and subsequent parkin recruitment which leads to polyubiquination of substrates and recruitment of LC3 adapters, p62, OPTN, NDP52, TAX1BP1 and NBR1. Interestingly, parkin polyubiquitination of MFN1 and MFN2 leads to their degradation and thus an imbalance towards mitochondrial fission, suggesting a link with mitophagy.^137^

The ubiquitin‐independent pathway recruits LC3 adapters either in the choline dehydrogenase (CHDH)/p62 complex or via TBC1D15/TBC1D17 complex interaction with FIS1 and LC3.^107^, ^137^ Alongside the canonical mitophagy mechanism, a more rapid contact between mitochondrial derived vesicles (MDVs) and lysosomes has been demonstrated through the formation of mitochondria–lysosome‐related organelles (MLRO) in the hepatocyte dedifferentiation process.^139^

Another mechanism suggested as quality control in the context of cellular hypoxia is the formation of megamitochondria secondary to an increase in the fusion/fission ratio. These megamitochondria are able to engulf and activate lysosomes with release of lysosomal proteases and activation of mitochondrial proteases, leading to a state of mitochondrial self‐digestion and ROS production.^138^

Alongside the quality control mechanisms discussed above, the interaction between lysosomes and mitochondria is essential for calcium, iron and lipid delivery to the mitochondria. Also, lysosomes of different sizes can contact multiple mitochondria at the same time.^140^

The lysosomal protein Rab7 is implicated in lysosomal dynamics and is postulated to regulate the tethering of lysosomes and mitochondria at contact sites via a hydrolysis mechanism, mediated by TBC1D15 and FIS1,^106^, ^140^ one of the mitochondrial fission factors, and is reportedly involved in the regulation of mitophagy^141^ (Figure 2). If Rab7 is not hydrolysed to the GDP bound form this leads to prolonged lysosomal–mitochondrial contact. Interestingly, Rab7 deficiency causes autosomal dominant Charcot–Marie–Tooth disease, type 2B (CMT2B disease, *RAB7*, MIM #602298).^142^

Another suggested regulator of mitochondria–lysosome contact site tethering is GDAP1, a glutathione S‐transferase on the OMM (Figure 2). Absence of GDAP1 leads to increased distance between lysosomes and mitochondria, decreased number of contact sites and reduced contact time. Pathogenic variants in *GDAP1* lead to autosomal dominant and autosomal recessive forms of axonal neuropathy (CMT2K, MIM #606598). The lysosomal equivalent of GDAP1 is suggested to be a lysosomal membrane protein, LAMP1. MFN2 seems to have an additional role in regulating mitochondrial–lysosomal tethering.^140^

As discussed earlier, calcium signalling is critical for mitochondrial function. Therefore, it is not surprising that there are redundant mechanisms for calcium delivery to the mitochondria. In fact, direct calcium influx into mitochondria has been demonstrated at lysosome–mitochondria contact sites. This seems to be mediated by TRPML1, encoded by *MCOLN1*, on the lysosomal side and VDAC1 and MCU in the OMM and IMM respectively^136^, ^140^ (Figure 2).

**MCOLN1 deficiency** (*MCOLN1*, autosomal recessive inheritance, MIM #605248) causes the lysosomal storage disorder Mucolipidosis type IV, where both lysosomal and mitochondrial dysfunction have been demonstrated.^136^, ^140^

Concerning lipid transfer, Niemann Pick C2 (NPC2) on the lysosomal side is involved in regulation of cholesterol trafficking towards the mitochondria at mitochondrial–lysosome contact sites. StAR‐related lipid transfer domain‐3 (STARD3) is postulated to regulate the contact sites to enhance cholesterol transfer, whereas MLN64, a steroidogenic acute regulatory protein‐related lipid transfer (START) domain‐containing protein localized to endolysosomal membranes, is reported to actively transfer cholesterol to the mitochondria.^140^

### 4.5 Mitochondria–nucleus interactions

The communication between the nucleus and mitochondria is bidirectional and is essential for mediating the response to stress triggers. This is an extremely complex area, and a detailed discussion of mitochondrial–nuclear crosstalk is beyond the scope of this review. We will only provide few examples of this interaction. The reader is referred to recent reviews on this topic for a more detailed discussion.^143^, ^144^

Nuclear‐respiratory factor 1 (NRF1) and nuclear respiratory factor 2 (NRF2), peroxisome proliferator‐activated receptors (PPARs), mitochondrial transcription factor A (Tfam), uncoupling protein 2 (UCP2), oestrogen‐related receptor (ERR) and PPARγ co‐activator 1α (PGC‐1α) are commonly recognised as components of the anterograde flux, from nucleus to mitochondria. Nuclear‐encoded mitochondrial microRNAs (mitomiRNAs) have also been suggested to regulate mitochondrial gene expression.^107^ NRF2 is a transcription factor of genes coding for enzymes with antioxidant functions.^145^

The retrograde flux, from mitochondria to the nucleus, is likely activated in the context of mitochondrial oxidative, metabolic or respiratory stress. Several mitochondrially derived peptides, as well as ROS, inorganic phosphate components, NAD and NADH and calcium are believed to play a role in the signalling. Interestingly, mitomiRNAs were found to be localised in the nucleus at the time of mitochondrial dysregulation and diseases, suggesting a possible duplex direction of these mitomiRNAs, even though the mechanism for their entrance into the nucleus is still to be clarified.^107^

Another component that is hypothesised to move in both directions depending on the trigger is ATFS‐1. ATFS‐1 is a transcription factor that moves into the mitochondria in conditions of mitochondrial Unfolded Protein Response (UPR) and to the nucleus to regulate the transcription of OXPHOS related genes in conditions of mitochondrial stress.^146^

The monooxygenase CLK‐1 is a mitochondrial enzyme involved in respiration. However, a nuclear isoform mediates a retrograde signal in response to mitochondrial ROS.^146^ FKBP51 is a HSP90‐binding immunophilin and it is localised in the mitochondria of every cell. Nevertheless, under oxidative or inflammatory stress it enters the nucleus where it probably plays a role as an antiapoptotic factor.^147^ In conditions of DNA damage, mitochondrial cytochrome *c* translocates to the nucleolus and plays a role in reducing p53 degradation, hence regulating nucleolar function and DNA repair pathway activation.^148^

Lonp1 belongs to the mitochondrial network and is localised in the cristae, being involved in mtDNA maintenance. This protein has been reported to also localise to the nucleus and increase its concentration there as part of the Heat Shock response, suggesting a strong link between mitochondria and the nucleus and a potential translocation of the protein from the mitochondria to the nucleus.^146^

**LONP1 deficiency** (*LONP1*, autosomal recessive inheritance, MIM #605490) causes Cerebral Ocular Dental Auricular Skeletal Anomalies Syndrome (CODAS), a complex multisystemic and developmental disorder.^149^

## 5 CONCLUSION

There is a complex interplay between mitochondrial lipid biosynthesis, membrane remodelling, cristae organization, protein translocation, protein synthesis and mitochondrial dynamics. None of these processes occurs in isolation, thus it is not surprising that some proteins have dual roles in different mitochondrial processes, and that there is phenotypic overlap between disorders of mitochondrial lipid biosynthesis, cristae organisation, proteostasis and mitochondrial dynamics. Mitochondrial lipids play important roles in interactions with other organelles, and we predict that defects of mitochondrial membrane contact sites will be increasingly recognised as a cause of human disease.

## AUTHOR CONTRIBUTIONS

**Martina Messina**: Conceptualising the content and the structure, drafting of manuscript, and conceptualisation and creation of figures. **Frédéric M. Vaz**: Manuscript review and editing, and figure creation. **Shamima Rahman**: Conceptualising the content, drafting and editing of the manuscript, conceptualisation and editing of figures.

## FUNDING INFORMATION

Martina Messina and Shamima Rahman acknowledge funding from Horizon Medicine and Innovate UK for the Recon4IMD project. Shamima Rahman acknowledges grant funding from Great Ormond Street Hospital Children's Charity, the Lily Foundation and the National Institute of Health Research (NIHR) Great Ormond Street Hospital Biomedical Research Centre. The views expressed are those of the authors and not necessarily those of the NHS, the NIHR, or the United Kingdom Department of Health. Frederic Maxime Vaz has no financial support to declare relevant to this study. The authors confirm independence from the funders. The content of the article has not been influenced by the funders.

## CONFLICT OF INTEREST STATEMENT

Martina Messina declares no competing interests. Frederic Maxime Vaz declares no competing interests. Shamima Rahman declares that she is an editor‐in‐chief of the Journal of Inherited Metabolic Disease and has provided consultancy on primary mitochondrial diseases to pharmaceutical companies as listed in the IJCME conflict of interest form.

## ETHICS STATEMENT

This article does not contain any studies with human or animal subjects performed by the any of the authors, hence no ethics approval is required.

## PATIENT CONSENT

This article does not contain any studies with human subjects performed by the any of the authors, hence no patient consent is required.

## ANIMAL RIGHTS

This article does not contain any studies with animal subjects performed by the any of the authors.

## Data Availability

All relevant data are included in the manuscript. No additional data are applicable.
